# Perinatal Outcomes After the Use of Kampo Medicines Containing Ephedra During Pregnancy

**DOI:** 10.1002/pds.70251

**Published:** 2025-11-03

**Authors:** Aoi Noda, Ryutaro Arita, Taku Obara, Minoru Ohsawa, Satoko Suzuki, Ken Haneda, Ryo Obara, Kei Morishita, Genki Shinoda, Keiko Murakami, Masatsugu Orui, Mami Ishikuro, Akiko Kikuchi, Shin Takayama, Tadashi Ishii, Shinichi Kuriyama

**Affiliations:** ^1^ Tohoku Medical Megabank Organization Tohoku University Sendai Japan; ^2^ Department of Molecular Epidemiology, Graduate School of Medicine Tohoku University Sendai Japan; ^3^ Department of Pharmaceutical Sciences Tohoku University Hospital Sendai Japan; ^4^ Department of Kampo and Integrative Medicine, Graduate School of Medicine Tohoku University Sendai Japan; ^5^ Department of Education and Support for Regional Medicine Tohoku University Hospital Sendai Japan; ^6^ Department of Obstetrics and Gynecology Tohoku University Hospital Sendai Japan; ^7^ International Research Institute of Disaster Science Tohoku University Sendai Japan

**Keywords:** birth cohort, herbal medicine, japanese traditional medicine, Kampo medicine, medication use, perinatal outcome, pregnant women

## Abstract

**Purpose:**

Japanese traditional (Kampo) medicines containing ephedra are used to relieve symptoms of the common cold during pregnancy. The risks associated with perinatal outcomes, however, remain unclear. Thus, we evaluated the risk of adverse perinatal outcomes associated with the use of Kampo medicines containing ephedra during pregnancy using data from the Tohoku Medical Megabank Project Birth and Three‐Generation Cohort Study (TMM BirThree Cohort Study).

**Methods:**

Questionnaires were distributed to pregnant women who participated in the TMM BirThree Cohort Study at approximately weeks 12 and 26 of pregnancy. Adverse perinatal outcomes in women who used Kampo medicines containing ephedra or acetaminophen during pregnancy were assessed. Odds ratios (ORs) were estimated using weighted logistic regression analyses.

**Results:**

Among 20 083 pregnant women, acetaminophen and Kampo medicines containing ephedra were used by 5.3% and 3.5% of women, respectively. The OR for caesarean section was 0.95 (95% confidence interval [CI], 0.75–1.20), for preterm birth (PTB) was0.99 (95% CI, 0.63–1.55), for low birth weight (LBW) was 1.04 (95% CI, 0.72–1.49), for small for gestational age (SGA) was 0.98 (95% CI, 0.58–1.65), and for low Apgar scores at 5 min was 0.85 (95% CI, 0.25–2.93) in the women who used Kampo medicines containing ephedra during pregnancy.

**Conclusions:**

No statistically significant association was seen between the use of Kampo medicines containing ephedra during pregnancy and an increased risk of needing a caesarean section, PTB, LBW, SGA, or low Apgar scores. Although further research is needed, this study may assist in clinical decision‐making.

**Summary**

- This is the first study to assess the risk of adverse perinatal outcomes associated with the use of Kampo medicines containing ephedra during pregnancy.
- Data were obtained from the Tohoku Medical Megabank Project Birth and Three‐Generation Cohort Study, a large Japanese birth cohort.
- Acetaminophen and Kampo medicines containing ephedra were used by 5.3% and 3.5% women, respectively.
- Among women who used Kampo medicines containing ephedra or acetaminophen during pregnancy, the prevalence of cesarean section, preterm birth, low birth weight, small for gestational age, and low Apgar scores at 5 min was 23.0%, 5.6%, 8.3%, 3.9%, and 0.7%, or 24.2%, 5.1%, 7.6%, 3.6%, and 0.7%, respectively.
- No statistically significant association was seen between the use of Kampo medicines containing ephedra during pregnancy and an increased risk of adverse perinatal outcomes.

## 1 Introduction

Herbal medicines are used by some women during pregnancy [1, 2, 3]. According to a systematic review of 111 studies across 51 countries, 34.4% of women used herbal medicines during pregnancy [4]; however, there are distinct differences in the reasons for using herbal medicines across various regions [3]. These may be pregnancy‐related, such as nausea and vomiting, or related to general health conditions, such as cold or skin problems [5]. Japanese traditional (Kampo) medicine originates from traditional Chinese medicine and is widely used in combination with various natural products with complementary medicinal properties [6, 7, 8, 9, 10, 11]. Kampo medicine is widely used in obstetrics in Japan [12, 13, 14, 15]. A previous study in Japan reported that Kampo medicines are prescribed to 45% of pregnant women during the prenatal and postpartum periods [16]. Additionally, more than 10% of pregnant women use Kampo medicines from the prenatal to second trimesters of pregnancy [17]. Other studies also showed that Kampo medicines are mainly used to relieve symptoms of colds and pregnancy‐related health ailments, such as nausea during pregnancy [3, 16, 17, 18]. In particular, kakkonto and shoseiryuto, which contain ephedra are most frequently used during pregnancy [8, 17]. Ephedra is a botanical herb used in traditional Chinese medicine and contains ephedrine and pseudoephedrine as its main alkaloids [19]. Ephedrine should be used with caution during pregnancy because it promotes perspiration, which impairs peripheral circulation and causes hemodynamic disturbances in the fetoplacental system [19, 20]. We previously reported that there was no significant association between the use of Kampo medicines containing ephedra during the first trimester of pregnancy and congenital malformations [21]. However, there is a lack of data on the potential risks associated with the use of Kampo medicines containing ephedra and perinatal outcomes, such as caesarean section, preterm birth (PTB), low birth weight (LBW), small for gestational age (SGA), and low Apgar score. Therefore, this study evaluated the risk of adverse perinatal outcomes associated with exposure to Kampo medicines containing ephedra during pregnancy, using data from the Tohoku Medical Megabank Project Birth and Three‐Generation Cohort Study (TMM BirThree Cohort Study), a multigenerational genome and birth cohort study.

## 2 Materials and Methods

### 2.1 Study Design

This study was based on data obtained from the TMM BirThree Cohort Study, a prospective cohort study conducted in the Miyagi Prefecture in Japan. Detailed information regarding the TMM BirThree Cohort Study has been previously provided [22, 23]. Pregnant women and their family members were contacted in obstetric clinics or hospitals between 2013 and 2017. In total, 23 730 pregnant women–infant pairs participated in the study. Written informed consent was obtained from all participants. All participants were free to decline their consent to participate in the study and were informed that there were no disadvantages or risks involved in their refusal to participate. The eligibility criteria for participants (expectant mothers) were as follows: (1) they should reside in the study area at the time of recruitment, and (2) they should be able to comprehend Japanese and complete the self‐administered questionnaire. The TMM BirThree Cohort Study protocol was approved by Tohoku University and the Internal Review Board of the Tohoku Medical Megabank Organization (2013–1–103‐1). This study followed the Strengthening the Reporting of Observational Studies in Epidemiology (STROBE) reporting guidelines for observational studies.

### 2.2 Data Collection

#### 2.2.1 Medication

All data on medication use during early and middle pregnancy were obtained using a paper‐based questionnaire distributed when the women were in approximately weeks 12 and 26 of pregnancy. Most of the questionnaires were distributed and collected by trained genome medical research coordinators through face–to–face interviews with pregnant women in approximately 50 obstetric clinics and hospitals in Miyagi, which participated in the recruitment process [24]. The questions were open‐ended. We collected data from the following three periods: (1) 12 months before pregnancy diagnosis (before pregnancy diagnosis), (2) the period between pregnancy diagnosis and approximately week 12 of pregnancy (from diagnosis to week 12), and (3) from approximately week 12 of pregnancy (post‐week 12 of pregnancy). We excluded those with missing acquisition time data. During periods (1) and (2), data on medication use were collected through the first questionnaire using the following question: “Have you used any medicines, supplements, or health foods from one year before pregnancy until you found out you were pregnant, or from when you found out you were pregnant until now? This includes patches, ointments, inhalants, vaccinations, etc. Please write the product names of any medicines, supplements, or health foods you are using. You may also attach copies of the medicine instructions or your medication record.” During period (3), data on medication use were collected through the second questionnaire using the following question: “Have you used any medicines, supplements, or health foods from the last survey to the present? This includes patches, ointments, inhalants, vaccinations, etc. Please write the product names of any medicines, supplements, or health foods you are using. You may also attach copies of the medicine instructions or your medication record.” The names of the medicines self‐reported by the participants were often ambiguous; therefore, determining the exact medications was difficult. Multiple pharmacists and one medical doctor matched the self‐reported names of medicines with generic names based on the Kyoto Encyclopedia of Genes and Genomes (KEGG) medicus [25]. The prevalence of the use of acetaminophen and Kampo medicines containing ephedra during pregnancy was evaluated. Next, medication data from the time of pregnancy diagnosis to approximately week 12 were used to evaluate the risk of adverse perinatal outcomes associated with exposure to Kampo medicines containing ephedra during pregnancy. We defined the Kampo medicines that contain ephedra and are commonly used to relieve symptoms of the common cold as follows: kakkonto (KEGG ID: D06698), kakkontokasenkyushin'i (D06928), shoseiryuto (D06987), maoto (D07042), and maobushisaishinto (D07043). In the 18th Japanese Pharmacopoeia, ephedra is defined as the terrestrial stem of *Ephedra sinica* Stapf, *Ephedra intermedia* Schrenk et C.A. Meyer, or *Ephedra equisetina* Bunge (Ephedraceae). Ephedra contains ephedrine and pseudoephedrine as ≥ 0.7% of its total alkaloids, calculated on the basis of dried material [26].

#### 2.2.2 Perinatal Outcomes

Data on perinatal outcomes were obtained from medical records at the time of childbirth. We also collected the medical information of participants who gave birth outside the study area owing to relocation during their pregnancy. We asked them to fill out a questionnaire to collect information equivalent to those obtained from medical records, such as the progress of pregnancy and birth and the child's one‐month checkup. We also visited the hospital where the child was born as necessary to collect information from the medical records. Caesarean section, PTB, LBW, SGA, and low Apgar scores (< 7) were evaluated. PTB was defined as the birth of a baby before 37 completed weeks of pregnancy. LBW was defined as a birth weight of less than 2500 g. SGA was defined as a weight below the 10th percentile for the gestational age.

#### 2.2.3 Covariates

Considering previous studies and the characteristics of the population in the present study, we included the pregnant women's ages at delivery, pregnancy complications, infertility treatments, delivery histories, smoking during pregnancy, alcohol use during pregnancy, body mass indices before pregnancy, medical history, annual household incomes, and educational levels as covariates for the perinatal outcomes [27, 28, 29, 30, 31, 32, 33]. Data on the pregnant women's ages, infertility treatments, and delivery histories were obtained from the medical records at registration for this cohort study. Data on pregnancy complications were obtained from postpartum medical records. The pregnancy complications in this study were defined as follows: threatened abortion, threatened premature delivery, fetal growth restriction, blood type incompatibility, gestational diabetes mellitus, premature rupture of the membranes, low‐lying placenta, deep vein thrombosis, hypertensive disorders of pregnancy, placental abruption, non‐reassuring fetal status, iron deficiency anemia, placenta previa, placenta accreta, uterine inversion, oligohydramnios, polyhydramnios, amniotic fluid embolism, intrauterine infection, atonic bleeding, hemolysis, elevated liver enzymes, low platelet count syndrome, twin‐to‐twin transfusion syndrome, and obstetrical disseminated intravascular coagulation. Gestational weeks and infant sexes were obtained from the medical records of the newborns. Data on household income, educational levels, medical history, alcohol consumption, and smoking status during pregnancy were obtained using self‐reported questionnaires. Pregnant women reported their smoking status in the first questionnaire as follows: (1) never smoked, (2) stopped before becoming aware of pregnancy, (3) stopped after becoming aware of pregnancy, and (4) smoked during early pregnancy. We divided the participants into two categories, with those answering (1) being categorized as without a history of smoking, and those answering (2), (3), or (4) as those with a history of smoking. In addition, pregnant women reported their drinking status in the first questionnaires as follows: (1) Drinking during early pregnancy, (2) former drinking, (3) never drinking, and (4) cannot drink because of constitution. For analysis, we further grouped those who answered with (1) and (2) as those with a history of alcohol consumption, and those who answered with (3) and (4) as those without a history of alcohol consumption. The medical histories are shown in Table S1. Dummy variables were created for missing data on educational level, annual household income, and medical history. Other missing data were excluded.

### 2.3 Statistical Analysis

Evaluating specific infectious agents is difficult in observational studies, leaving an opportunity for uncontrolled confounding factors. To eliminate the possibility of confounding by infectious diseases and to adjust for the same exposure conditions, women who used acetaminophen, which is used to relieve symptoms of the common cold and is considered safe during pregnancy [34], were included in the reference group. The characteristics of the pregnant women and infants were compared between two groups: pregnant women who used acetaminophen and those who used Kampo medicines containing ephedra. We excluded any participants who used both Kampo medicine containing ephedra and acetaminophen. Continuous and categorical variables were described as mean (standard deviation), frequency, or proportion. To assess the baseline balance between groups, both *P* values (calculated using the two‐sided chi‐square test) and standardized mean differences (SMDs) were used. Statistical significance was set at *p* < 0.05. SMDs were calculated for continuous, binary, and categorical variables, with values > 0.1 considered indicative of imbalance. We constructed a propensity score to balance the observed baseline characteristics based on the Inverse Probability Treatment Weighting (IPTW). The propensity scores were calculated based on the pregnant women's ages at delivery, pregnancy complications, infertility treatments, delivery histories, smoking during pregnancy, alcohol use during pregnancy, body mass index before pregnancy, annual household incomes, education levels, and medical history using a logistic regression model. Weighted logistic regression analyses were performed to determine the association between the use of Kampo medicines containing ephedra during pregnancy and the risk of adverse perinatal outcomes. Odds ratios (ORs) and confidence intervals (CIs) were calculated. Sensitivity analyses were conducted to assess the adverse perinatal outcomes. First, the analyses were repeated for those who used Kampo medicines containing ephedra, including those who used them for illnesses other than the common cold during pregnancy. In addition to the five Kampo medicines containing ephedra, which are used to relieve symptoms of the common cold, we evaluated other Kampo medicines containing ephedra, including eppikajutsuto (D06921), kakkontokajutsubuto (D06927), keishakuchimoto (D06951), keimakakuhanto (D06953), gokoto (D06955), goshakusan (D06956), shimpito (D06994), bofutsushosan (D07041), makyokansekito (D07044), makyoyokukanto (D07045), and yokuininto (D07048). These Kampo medicines are mainly used for asthma, arthralgia, obesity, and edema but not for the common cold [35, 36, 37, 38, 39, 40, 41, 42]. Second, the analyses were repeated for those who used Kampo medicines containing ephedra in the second trimester. Statistical analyses were performed using SAS v.9.4 (SAS Institute Inc., Cary, NC, USA).

## 3 Results

### 3.1 Characteristics of the Participants

**FIGURE 1:**
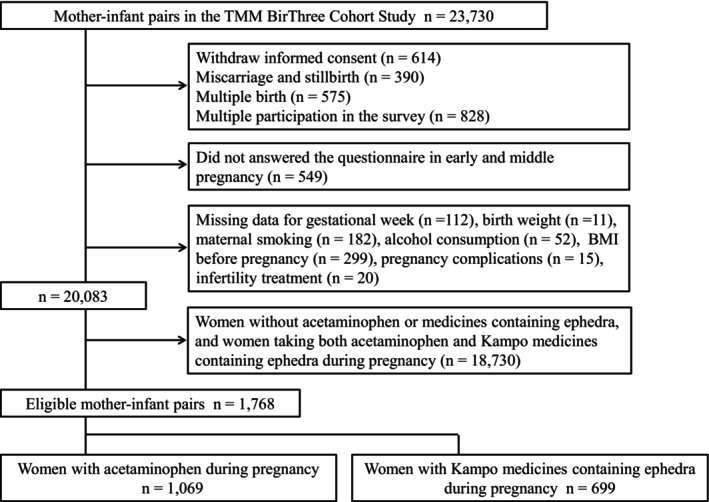
Flow chart for the selection of participants for the study.

Of the 23 730 pregnant women–infant pairs, 614 women withdrew informed consent, 390 had miscarriages and stillbirths, 575 had multiple births, and 828 participated more than once because they became pregnant multiple times during the recruitment period. Only the first pregnancy of each woman was included in this analysis, and data from subsequent pregnancies were excluded. In addition, 549 did not answer the questionnaire during early and middle pregnancy. These individuals were excluded from the study (Figure 1). We further excluded women who used both acetaminophen and Kampo medicines containing ephedra during pregnancy. Data from 1768 eligible pregnant women–infant pairs were analyzed, as shown in the flowchart in Figure 1. Of the 1768 women, 1069 (5.3%) used acetaminophen and 699 (3.5%) used Kampo medicines containing ephedra during pregnancy. The maternal and adverse perinatal outcomes characteristics are shown in Table 1. The male proportions were 51.3% (*n* = 548) and 52.4% (*n* = 366) in women who used acetaminophen and in those who used Kampo medicines containing ephedra, respectively. The women who used Kampo medicines containing ephedra during pregnancy were older, more highly educated, and less likely to have pregnancy complications than those who used acetaminophen. Information on the frequency of use of each Kampo medicine containing ephedra and the sources of acquisition is shown in Table 2.

**TABLE 1:** Characteristics of the pregnant women who used Kampo medicines during pregnancy.

	Total		Women with acetaminophen		Women with Kampo medicines containing ephedra		*P*	SMD
	*n* = 1768		*n* = 1069		*n* = 699			
Maternal characteristics								
Pregnant woman's age at delivery, mean (SD), y	32.7	(4.7)	32.4	(4.8)	33.1	(4.6)	0.001	0.16
BMI before pregnancy, *n*, %								
< 18.5 kg/m^2^	197	11.1	128	12.0	69	9.9	0.013	−0.07
18.5 — < 25.0 kg/m^2^	1304	73.8	762	71.3	542	77.5		0.14
≥ 25.0 kg/m^2^	267	15.1	179	16.7	88	12.6		−0.12
Alcohol use at pregnancy, *n*, %								
Drinking at early pregnancy	369	20.9	217	20.3	152	21.8	0.810	0.04
Former	644	36.4	395	37.0	249	35.6		−0.03
Never	665	37.6	400	37.4	265	37.9		−0.13
Cannot drink because of constitution	90	5.1	57	5.3	33	4.7		−0.1
Smoking at pregnancy, *n*, %								
Never	1041	58.9	601	56.2	440	63.0	0.004	0.14
Stopped before pregnancy	463	26.2	285	26.7	178	25.5		−0.03
Stopped after pregnancy	229	13.0	156	14.6	73	10.4		−0.13
Smoking during early pregnancy	35	2.0	27	2.5	8	1.1		−0.10
Pregnancy complications, *n*, %	846	47.9	547	51.2	299	42.8	0.001	0.17
Infertility treatment, *n*, %								
Natural pregnancy	1639	92.7	988	92.4	651	93.1	0.575	0.03
Assisted conception	129	7.3	81	7.6	48	6.9		−0.03
Medical history, *n*, %								
0 or 1	582	32.9	336	31.4	246	35.2	0.205	0.08
≥ 2	616	34.8	375	35.1	241	34.5		−0.01
Missing	570	32.2	358	33.5	212	30.3		−0.07
Delivery history, *n*, %	995	56.3	574	53.7	421	60.2	0.007	0.13
Socioeconomic status								
Educational level, *n*, %								
High school graduate or less	371	21.0	245	22.9	126	18.0	0.003	−0.12
Junior or vocational college graduate	461	26.1	266	24.9	195	27.9		0.07
University graduate or above	355	20.1	192	18.0	163	23.3		0.13
Missing	581	32.9	366	34.2	215	30.8		−0.07
Annual household income, *n*, %								
< 4 000 000 yen	552	31.2	351	32.8	201	28.8	0.186	−0.09
4 000 000 – 6 000 000 yen	553	31.3	337	31.5	216	30.9		−0.01
6 000 000 yen <	584	33.0	335	31.3	249	35.6		0.09
Missing	79	4.5	46	4.3	33	4.7		0.02
Adverse perinatal outcomes								
Caesarean section, *n*, %	420	23.8	259	24.2	161	23.0	0.564	−0.03
Birth weight, mean (SD), (g)	3045.9	(424.4)	3050.1	(432.0)	3039.5	(404.8)	0.605	−0.03
Birth weight, *n*, %								
≥ 2500 g	1629	92.1	988	92.4	641	91.7	0.582	−0.03
< 2500 g	139	7.9	81	7.6	58	8.3		0.03
Gestational week, *n*, %								
≥ 37	1675	94.7	1015	95.0	660	94.4	0.627	−0.02
< 37	93	5.3	54	5.1	39	5.6		0.02
SGA, *n*, %	65	3.7	38	3.6	27	3.9	0.737	0.02
Apgar score at 1 min < 7, *n*, %	51	2.9	29	2.7	22	3.2	0.594	0.03
Apgar score at 5 min < 7, *n*, %	12	0.7	7	0.7	5	0.7	0.227	0.01

Abbreviations: BMI, body mass index; SD, standard deviation; SMD, standardized mean difference.

**TABLE 2:** The frequency of Kampo medicines containing ephedra during pregnancy (*n* = 699).

(a) From diagnosis to week 12 (*n* = 389)		
General name	*n*	%
Shoseiryuto	205	11.60
Kakkonto	165	9.33
Gokoto	14	0.79
Kakkontokasenkyushin'i	10	0.57
Bofutsushosan	5	0.28
Maoto	4	0.23
Maobushisaishinto	4	0.23
Eppikajutsuto	3	0.17
Makyokansekito	3	0.17
Shimpito	1	0.06

(b) Post‐week 12 of pregnancy (*n* = 432)		
General name	*n*	%
Shoseiryuto	271	15.33
Kakkonto	139	7.86
Gokoto	10	0.57
Kakkontokasenkyushin'i	8	0.45
Shimpito	5	0.28
Maobushisaishinto	5	0.28
Maoto	3	0.17
Bofutsushosan	2	0.11
Makyokansekito	2	0.11
Yokuininto	1	0.06
Eppikajutsuto	1	0.06
Keishakuchimoto	1	0.06

*Note:* Obtained via prescription (*n* = 363, 87.7%), other sources (*n* = 43, 10.3%), or unknown (*n* = 8, 1.9%). Obtained via prescription (*n* = 420, 93.8%), other sources (*n* = 25, 5.6%), or unknown (*n* = 8, 0.6%).

### 3.2 Adverse Perinatal Outcomes Associated With Exposure to Kampo Medicines Containing Ephedra During Pregnancy

The prevalence of adverse perinatal outcomes was similar between women who used Kampo medicines containing ephedra and those who used acetaminophen. Specifically, the rates of caesarean section (23.0% vs. 24.2%), PTB (5.6% vs. 5.1%), LBW (8.3% vs. 7.6%), SGA (3.9% vs. 3.6%), and low Apgar scores at 5 min (0.7% vs. 0.7%) did not markedly differ between the groups (Table 1). The adjusted ORs for these outcomes were close to unity and not statistically significant (Table 3). For example, the adjusted ORs were 0.95 (95% CI, 0.75–1.20) for the caesarean section, 0.99 (95% CI, 0.63–1.55) for PTB, and 1.04 (95% CI, 0.72–1.49) for LBW.

**TABLE 3:** Association between the use of Kampo medicines containing ephedra during pregnancy and pregnancy outcome risks. (*n* = 699).

			Crude			Adjusted model 1			Adjusted model 2		
			Odds ratio	95% CI		Odds ratio	95% CI		Odds ratio	95% CI	
	*n*	%		Lower	Upper		Lower	Upper		Lower	Upper
Caesarean section	161	23.0	0.94	0.75	1.17	0.90	0.71	1.12	0.95	0.75	1.20
PTB	39	5.6	1.11	0.73	1.70	1.11	0.73	1.70	0.99	0.63	1.55
LBW	58	8.3	1.10	0.78	1.57	1.09	0.77	1.55	1.04	0.72	1.49
SGA	27	3.9	1.09	0.66	1.80	1.10	0.66	1.82	0.98	0.58	1.65
Low Apgar score at 1 min (< 7)	22	3.2	1.17	0.66	2.05	1.16	0.66	2.04	1.01	0.56	1.83
Low Apgar score at 5 min (< 7)	5	0.7	1.09	0.35	3.46	1.15	0.36	3.64	0.85	0.25	2.93

*Note:* Women who used acetaminophen during pregnancy were taken as reference in this analysis. (*n* = 1069). Model 1: Adjusted for maternal age at delivery. Model 2: We constructed a propensity score based on the IPTW. The propensity score of using Kampo medicines containing ephedra or acetaminophen during pregnancy were calculated using the pregnant women's ages at delivery, pregnancy complications (threatened abortion, threatened premature delivery, fetal growth restriction, blood type incompatibility, gestational diabetes mellitus, premature rupture of the membranes, low‐lying placenta, deep vein thrombosis, hypertensive disorders of pregnancy, placental abruption, non‐reassuring fetal status, iron deficiency anemia, placenta previa, placenta accreta, uterine inversion, oligohydramnios, polyhydramnios, amniotic fluid embolism, intrauterine infection, atonic bleeding, hemolysis, elevated liver enzymes, low platelet count syndrome, twin‐to‐twin transfusion syndrome, obstetrical disseminated intravascular coagulation), infertility treatments, delivery histories, smoking during pregnancy, alcohol use during pregnancy, body mass indices before pregnancy, annual household incomes, educational levels, and medical history (hepatitis B, hepatitis C, stomach cancer/gastric cancer, colorectal cancer, lung cancer, liver cancer, kidney cancer, pancreatic cancer, skin cancer, breast cancer, testicular cancer, prostate cancer, brain tumor, leukemia, malignant lymphoma, multiple myeloma, ovarian cancer, cervical cancer, endometrial cancer, iron‐deficiency anemia, type 1 diabetes, type 2 diabetes, hyperthyroidism/Graves' disease, hypothyroidism/Hashimoto's disease, hyperuricemia/gout, hyperlipidemia, depression (before disaster), depression (after disaster), dementia, bipolar disorder, anxiety disorder, PTSD (pre‐disaster), PTSD (post‐disaster), Schizophrenia, attention‐deficit/hyperactivity disorder (ADHD), learning disability (LD), pervasive developmental disorders (incl. autism, Asperger's), anorexia nervosa, bulimia nervosa, Parkinson's disease, epilepsy, migraine, meningitis/encephalitis, hydrocephalus, glaucoma, macular degeneration, corneal disease, hearing loss, allergic conjunctivitis, hypertension, cerebral hemorrhage, cerebral infarction, subarachnoid hemorrhage, myocardial infarction/angina pectoris, aneurysm/aortic dissection, heart failure, Marfan syndrome, atrial fibrillation, pacemaker implantation, ventricular fibrillation (with defibrillator), allergic rhinitis, bronchial asthma, chronic bronchitis, chronic sinusitis, chronic obstructive pulmonary disease (COPD), gastroesophageal reflux disease (GERD), gastritis, gastric ulcer, duodenal ulcer, irritable bowel syndrome (IBS), Crohn's disease, ulcerative colitis, fatty liver, gallstones, pancreatitis, urticaria, contact dermatitis, atopic dermatitis, psoriasis, scoliosis, osteoarthritis of the knee, Kawasaki disease, collagen disease, autoimmune disease, systemic lupus erythematosus (SLE), rheumatoid arthritis, chronic nephritis (e.g., IgA nephropathy, glomerulonephritis), renal dialysis, nephrotic syndrome, ureteral or kidney stones, endometriosis, uterine fibroids, congenital heart disease).

Abbreviations: CI, confidence interval; LBW, low birth weight; PTB, preterm birth; SGA, small for gestational age.

In sensitivity analyses, the use of all Kampo medicines containing ephedra during pregnancy yielded results similar to those of the primary analyses (Table S3). Additionally, the use of Kampo medicines containing ephedra in the second trimester was not associated with the risk of adverse perinatal outcomes in infants compared with acetaminophen in the second trimester although elevated point estimates were observed (Table S4).

## 4 Discussion

We did not find a statistically significant association between the use of Kampo medicines containing ephedra during pregnancy and the risk of adverse perinatal outcomes. The results did not affect the conclusions of the sensitivity analyses. To the best of our knowledge, this is the first study to evaluate the risk of adverse perinatal outcomes associated with exposure to Kampo medicines containing ephedra during pregnancy in Japan.

Kampo medicines have been reported to be more acceptable to pregnant women [1, 43] because they are perceived as being natural and therefore free of risks [37]. However, there is currently limited scientific evidence supporting the safety of Kampo medicines in pregnant women [44, 45]. The association between most Kampo medicines and major congenital malformations (MCMs) has not been fully elucidated. Moreover, previous reports have shown that some herbs are associated with perinatal outcomes such as PTB, intrauterine growth, and LBW [5]. Clear evidence of the adverse effects of herbs on perinatal outcomes exists only in some cases; however, the data are not sufficient [46]. In addition, for other plants, advisory warnings are cautious because available data on their safety are limited and a cause–effect relationship has not yet been established [5].

In Japan, Kampo medicines for the common cold are used frequently during pregnancy [16, 18, 47]. These Kampo medicines often contain ephedra, which contains ephedrine and related alkaloids, increases blood pressure and heart rate, causes central nervous system activity, and stimulates the uterine muscle [38]. In 2004, the Food and Drug Administration published a final rule prohibiting the sale of dietary supplements containing ephedrine alkaloids (ephedra) [48]. Although ephedra is not prohibited in Japan, the packages of Kampo medicines containing ephedra state that this drug should be used in pregnant women or women who may possibly be pregnant only if the therapeutic benefits outweigh the possible risks associated with treatment [49]. We previously reported that the use of Kampo medicines containing ephedra was not associated with MCMs [21]. In this study, the association between the use of Kampo medicines containing ephedra during pregnancy and the risk of adverse perinatal outcomes was not statistically significant. The 95% CIs were wide, suggesting that the sample size might have been insufficient. In addition, it has been reported that the adverse effects of alkaloids, such as ephedrine, can be influenced by gastric pH [50, 51]. However, blood concentrations were not measured, and the effects of concomitant medications that could alter gastric pH could not be taken into account in this study. Kampo medicines are composed of multiple crude drugs and contain numerous active constituents. Due to their complex, multi‐component, and multi‐target nature, it remains challenging to suggest a clear pharmacological mechanism of action. However, given the limited information on the association between Kampo medicines containing ephedra and perinatal outcomes, it is important to evaluate the risks of caesarean section, PTB, LBW, SGA, and low Apgar scores, and further research is warranted.

In many countries, Kampo medicines are sold over the counter (OTC), which makes them easily accessible. A previous study has shown that more than half of pregnant women use OTC or supplements during pregnancy [52]. As patients may think that Kampo medicines are natural and free of any adverse effects, unlike conventional medicines, their use is not always reported to healthcare professionals [53]. One study reported that Kampo formulas prescribed by physicians are less harmful than OTC products [54]. Therefore, healthcare professionals should routinely inquire whether pregnant women are using Kampo medicines or other natural products. In this study, data on Kampo medicine use from other sources including OTC were collected through a self‐reported questionnaire. We found that approximately 10% of pregnant women used herbal medicines from other sources during early pregnancy, and 5%–6% used the medicines during mid‐pregnancy. These data allowed us to assess proper medication use among pregnant women.

This study had some limitations. First, although IPTW based on propensity scores is a widely used method for causal inference in observational studies, it has several inherent limitations. These include sensitivity to the misspecification of the propensity score model, the possibility of extreme weights that can increase the variance of the estimates, and the inability to account for unmeasured confounding. We could not clarify whether all adjustment variables used in the propensity score model, such as pregnancy complications and medical history, were measured before exposure. These factors should be considered when interpreting our findings. Moreover, this study was limited to pregnant women who voluntarily participated in the TMM BirThree Cohort Study. Therefore, cooperative and health‐conscious pregnant women were more likely to participate in the study. This study further did not provide information on the use of Kampo medicines during the third trimester. Therefore, further evidence on adverse perinatal outcomes of Kampo medicines containing ephedra is necessary. The findings of this study may, however, assist in clinical decision‐making. There also remains the potential for misclassification or misinterpretation of medication use because of the self‐reported questionnaire. However, this study included OTC Kampo medicines. This is a unique strength, because such an analysis would not be feasible using only prescription databases. Additionally, retrospective questionnaires are prone to recall bias. However, our results may be more valid because pregnant women are generally more concerned about the use of medications than the general population. We also did not consider the frequency or quantity of the medications used. A dosage is a very important factor in any medication. However, its details could not be captured in our study. Therefore, the findings of the study are inconclusive, and it would be desirable to administer Kampo medicines containing ephedra with expert‐guided dosage during pregnancy. The maximum daily dosage of ephedra may be generally low, with a limit of 5 g in Kampo medicine objected in this study. It is possible that shoseiryuto and maobushisaishinto were administered daily for allergic rhinitis. It was difficult to exclude the influence of viruses based on whether the participants were taking a particular drug, which may have introduced another bias. Some pregnant women who had miscarriages or stillbirths further did not return the questionnaires or withdrew their consent. Therefore, the use of medications in women whose pregnancies ended in abortion or stillbirth may not have been thoroughly evaluated.

## 5 Conclusion

In this study, there was no statistically significant association between the use of Kampo medicines containing ephedra during pregnancy and an increased risk of needing a caesarean section, PTB, LBW, SGA, pregnancy complications, or low Apgar scores (< 7). Although further research is needed, this study may assist in clinical decision‐making.

### 5.1 Plain Language Summary

Japanese traditional (Kampo) medicines containing ephedra are used to relieve symptoms of the common cold during pregnancy. The risks associated with perinatal outcomes, however, remain unclear. Thus, we evaluated the risk of adverse perinatal outcomes associated with the use of Kampo medicines containing ephedra during pregnancy using data from the Tohoku Medical Megabank Project Birth and Three‐Generation Cohort Study (TMM BirThree Cohort Study). Questionnaires were distributed to pregnant women who participated in the TMM BirThree Cohort Study at approximately weeks 12 and 26 of pregnancy. Adverse perinatal outcomes in women who used Kampo medicines containing ephedra or acetaminophen during pregnancy were assessed. Among 20 083 pregnant women, acetaminophen and Kampo medicines containing ephedra were used by 5.3% and 3.5% of women, respectively. The OR of caesarean section, preterm birth, low birth weight, small for gestational age, and low Apgar scores at 5 min were close to unity in the women who used Kampo medicines containing ephedra during pregnancy. No statistically significant association was seen between the use of Kampo medicines containing ephedra during pregnancy and an increased risk of adverse perinatal outcomes. This study may assist in clinical decision‐making for the common cold during pregnancy.

## Author Contributions

A.N. was responsible for conducting the study, analyzing the data, interpreting the results, and drafting the manuscript. T.O. and S.K. supervised this study. R.A., T.O., S.S., MOhsawa, and K.H. contributed to the interpretation of results and provided critical feedback. T.O., R.O., K.M., G.S., MOrui, K.M., M.I., A.K., S.T., T.I., and S.K. provided advice on the essential intellectual content and helped draft the manuscript. All authors have reviewed and approved the final manuscript.

## Ethics Statement

The TMM BirThree Cohort Study protocol was reviewed and approved by the Ethics Committee of the Tohoku University Tohoku Medical Megabank Organization (2013–1–103–1). Trained genome medical research coordinators were placed in each clinic, hospital, or community support center to provide information on the TMM BirThree Cohort Study to potential participants and to receive signed informed consent from those who agreed to participate in the study.

## Consent

The authors have nothing to report.

## Conflicts of Interest

R.A., MOhsawa, A.K., S.T., and T.I. belong to the Department of Kampo and Integrative Medicine at the Tohoku University School of Medicine. The department received a grant from Tsumura & Co. a Japanese manufacturer of Kampo medicine; however, the grant was used as per Tohoku University guidelines. Potential conflicts of interest were addressed and managed appropriately by the Tohoku University Benefit Reciprocity Committee. The authors declare that this study was conducted in the absence of any commercial or financial relationships that could be construed as potential conflicts of interest.

## Supporting information


**Table S1:** List of 93 health conditions defining medical history (*n* = 20 083).
**Table S2:** Characteristics of the excluded and included populations.
**Table S3:** Sensitivity analysis to evaluate the association between the use of all Kampo medicines containing ephedra during pregnancy (*n* = 734).
**Table S4:** Sensitivity analysis to evaluate the association between the use of Kampo medicines containing ephedra in the second trimester and the risk of pregnancy outcome (*n* = 415).

## Data Availability

Data obtained from the TMM BirThree Cohort Study were incorporated into the TMM Biobank. All data analyzed in the present study is available for research purposes, with the approval of the Sample and Data Access Committee of the Biobank.
